# Transmit Beamforming Design Based on Multi-Receiver Power Suppression for STAR Digital Array

**DOI:** 10.3390/s24020622

**Published:** 2024-01-18

**Authors:** Tairan Lin, Xizhang Wei, Jingtong Lai, Mingcong Xie

**Affiliations:** Department of Electronics and Communication Engineering, Sun Yat-sen University, Guangming District, Shenzhen 518107, China

**Keywords:** transmit beamforming, simultaneous transmit and receive, phased array, power amplifier, radiant power, multiple receivers

## Abstract

The simultaneous transmit and receive (STAR) array system provides higher radiation gain and data rate compared to traditional radio system. Because of the various mutual couplings between each pair of transmit and receive elements, it is a great challenge to suppress the incident self-interference power at multiple receive elements, which is usually much higher than the desired signal of interest (SoI) power and causes the saturation of receive links and the distortion of the digital SoI. In this paper, we propose an optimized method for transmit beamforming based on radiation power constraints and transmit power control. Through adaptive transmit beamforming, high isolation between the transmit array and each receive link is achieved, minimizing the self-interference power at each receiving element. This method effectively reduces the self-interference power, avoiding distortion of the SoI digital signal caused by limited-bit analog-to-digital converters (ADCs). Simulation results demonstrate that this optimized transmit beamforming method can achieve more than 100 dB effective isotropic isolation (EII) on a 32-element two-dimensional phased array designed in HFSS, reducing the maximum incident self-interference power at the receive channels by approximately 35 dB, while effectively controlling the attenuation of the transmit gain. We also present the advantages in receive subarray isolation and lower ADCs digits under the transmit ABF method.

## 1. Introduction

With the increasing competition for spectrum resources, simultaneous transmit and receive (STAR) technology has become quite popular in the past decade. Unlike Time Division Duplex (TDD) or Frequency Division Duplex (FDD) techniques [1], STAR aims to achieve simultaneous transmission and reception of signals at the same time, frequency, and system, which is known as in-band full-duplex (IBFD) in the communication field. The advancement of this technology will bring immeasurable benefits to the field of wireless communication, radar, and electronic warfare [2,3,4]. Its biggest challenge is to eliminate the impact of strong self-interference signals through the digital domain, analog domain, and propagation domain methods.

On the other hand, multiple antenna array systems can achieve flexible functionalities that are not available in single-antenna systems through narrow beam synthesis scanning. Array systems are widely used in various electronic radio fields [5], such as communication MIMO systems, phased array radars, and more. Implementing STAR technology on array systems holds infinite potential in the future. High data rate, high radiation gain, and multifunctional application can be implemented in a STAR array system.

**Figure 1:**
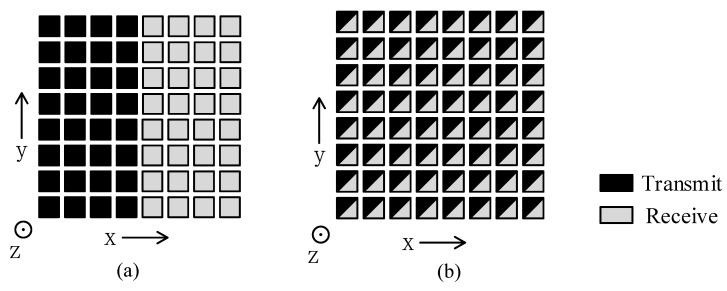
Two types of 64-element simultaneous transmit and receive arrays: (**a**) ALSTAR; (**b**) ELSTAR.

Generally, the simultaneous transmit and receive arrays can be divided into two types: aperture-level simultaneous transmit and receive (ALSTAR) and element-level simultaneous transmit and receive (ELSTAR) arrays [3], as shown in Figure 1.

In Figure 1a, the ALSTAR array divides the whole aperture into two separate sub-apertures for transmission and reception simultaneously. In Figure 1b, the ELSTAR array utilizes all elements of the entire aperture for simultaneous transmit and receive, either through the design of simultaneous transmit and receive single antennas or by employing a high-isolation circular coupler below each element. Compared to ALSTAR, ELSTAR doubles the area of the transmit and receive array, allowing for better utilization of the entire aperture and resulting in a narrower synthesized beam and higher gain. However, ELSTAR has higher system complexity and design difficulty. As for the receive array of this two-STAR array, the multi-receiver just means that each receive link can be regarded as an independent receiver, or it can be divided into multiple receive subarrays for different uses. Therefore, this kind of receive array has very high flexibility, and it is also widely used nowadays.

**Figure 2:**
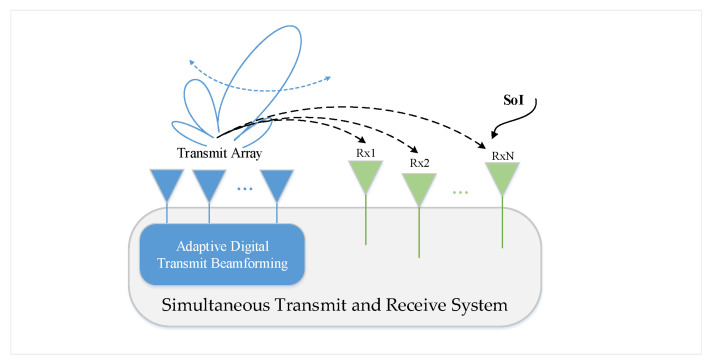
Adaptive transmit beamforming application in a multi-receiver STAR system.

Additionally, the beamforming of an array system can be implemented in analog or digital domains. In detail, analog beamforming is typically achieved using attenuators and phase shifters, while digital beamforming utilizes digital signal processing chips such as a field programmable gate array (FPGA) or a digital signal processor (DSP). The major challenge in achieving simultaneous transmit and receive is suppressing the strong self-interference signal at the receiving end (Rx) from the transmitting end (Tx). In a STAR array system, this challenge has been upgraded because of multiple coupling paths between every Tx and Rx. Overall, beamforming operations can bring significant benefits to the suppression of self-interference in simultaneous transmit and receive arrays. By appropriately designing beamforming weights, the array system can maximize signal gain in the desired directions for both transmission and reception, while minimizing the self-interference signal power at the Rx. The application scenario of this paper is shown in Figure 2.

Numerous studies have demonstrated the role of beamforming techniques in suppressing self-interference in full-duplex array systems [6,7,8,9,10,11,12,13,14,15,16,17,18,19]. Everett presented a digital-controlled method, called SoftNull, to enable full-duplex in many-antenna systems [6]. Chen studied FD phased arrays with joint transmit and receive beamforming with the objective of achieving improved FD data rates [7]. Xie and Hu used brainstorming optimization and genetic algorithm for the adaptive beamforming and sparse design of fully digital ALSTAR arrays [8,9,10]. Qiu adopted a linear constrained minimum variance algorithm to optimize the beamforming, which provides at least 110 dB of isolation for a 40 Tx × 40 Rx linear array [11,12]. Shi et al. presented a robust transmit beamforming SI cancellation optimization with maximum receive power constraints and formulated a worst-case uncertainty channel error model [13]. MIT Lincoln Laboratory presented an ALSTAR array model with observe channels and constructed an 8-channel ALSTAR array prototype, which achieves 140.5 dB effective isotropic isolation (EII) [14,15,16]. Furthermore, Cummings et al. used deep learning and iterative optimization for adaptive beamforming vectors based on the ALSTAR model they proposed [17,18], which achieved 187.1 dB effective isotropic isolation in a 25 Tx × 25 Rx array. Roberto studied full-duplex MIMO with finite-resolution phase shifters under hybrid beamforming, to maximize the spectral effciency and improve the isolation [19].

However, the aforementioned adaptive beamforming methods fail to effectively minimize the incident self-interference power at each receiving element while ensuring efficient control of the radiation power. In the case of fully digital phased arrays, there are several transmit links and receive links generally. Each receiving link is equipped with a finite-bit analog-to-digital converter (ADC). The whole receive array can be regarded as consisting of multiple digital receivers, and the digital signals on each link are allowed to be processed separately. Failure to reduce the incident self-interference may result in inadequate conversion of the relatively weaker signal of interest (SoI) into a digital signal. In prior studies on full-duplex or STAR systems, researchers commonly addressed this issue by employing RF self-interference cancellation (SIC) and antenna decoupling techniques [20,21,22]. But it is hard to use them with a STAR array system for a large number of the transmit and receive channels.

**Figure 3:**
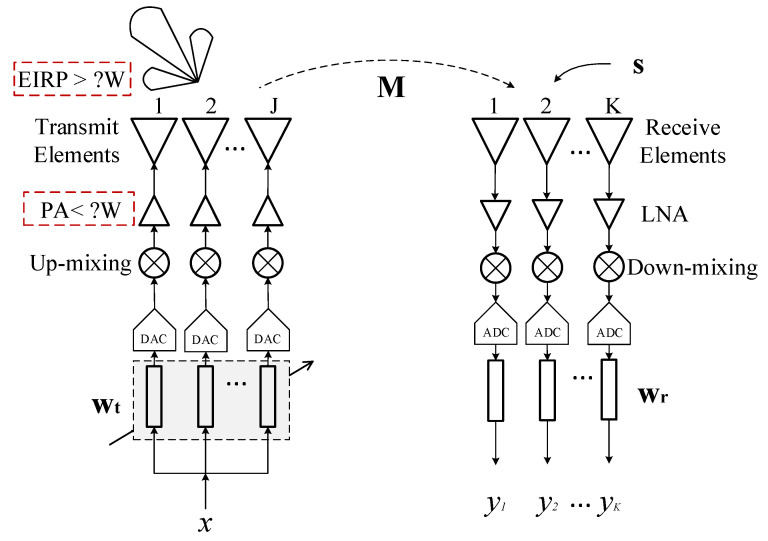
The simplified diagram of the STAR array system with the constraint of radiation power and transmit power in red dashed box.

In this paper, we propose an optimization method for transmit beamforming based on radiation power constraints. By employing adaptive transmit beamforming, the goal is to achieve high isolation between the transmit array and each receiving link, while minimizing the self-interference power at each receive element. Additionally, at the receiving antennas, the power of the desired SoI is often much smaller compared to that of the self-interference signal. This method effectively reduces the self-interference power at every receive element, not part of them, thereby avoiding distortion of the SoI digital signal caused by limited-bit analog-to-digital converters (ADCs). This means that the SoI incidenting to each receive channel can be digitalized successfully. The simplified diagram of the array system is shown in Figure 3.

From the analysis of Figure 3, we optimize a set of transmit beamforming vectors $w_{t}$ to satisfy the requirements of maximum output power of the power amplifier (PA) and minimum main beam radiation power (as shown in the red dashed box). More concretely, the effective isotropic radiated power (EIRP) is often used to describe the radiation power in the far-field desired direction.We modulate the transmitted signals to minimize the power reaching the receive elements, approaching the low power of the SoI, in order to prevent saturation of the receive link and distortion caused by the limited number of bits in the ADCs. For the effectiveness of SoI quantization, the uniform reduction of incident SI power on multiple receive elements can reduce the design difficulty of the receive array. In addition, the improved isolation between the transmit array and each receive element also allows each channel of the receive array to work flexibly and independently, rather than being only used as a phased array. In the scenario of a receive phased array, the digital receive beamforming vector $w_{r}$ in Figure 3 should also be considered certainly. Note that Figure 3 is suitable for describing both the ALSTAR and ELSTAR phased array systems, except for the use of circulators and a combination of antennas. In Section 3, the performance of ALSTAR is mostly shown and analyzed for more obvious observation. Table 1 lists all the acronyms and their definitions appearing in this paper.

## 2. System Model

### 2.1. STAR Array Architecture

**Figure 4:**
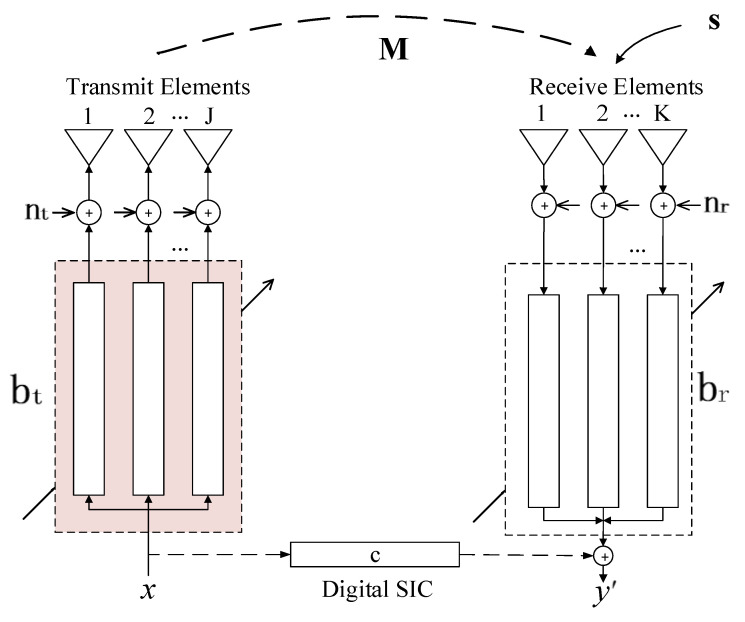
The signal flow diagram of a STAR digital array system. The transmit beamforming part that we study is marked in a red box.

Figure 4 shows the signal flow diagram of a STAR digital array system, with J and K representing the number of transmitting and receiving elements, respectively.

**Figure 5:**
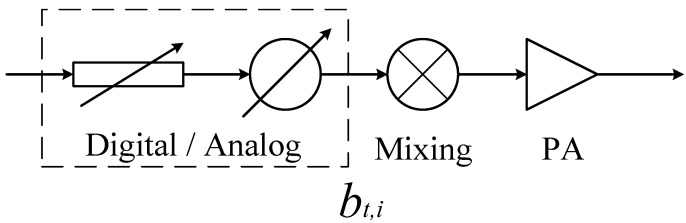
The components meaning of the symbol $b_{t}$. As for the amplitude and phase controller in the dashed box, we can use FPGA to calculate by digital accurately, or an analog attenuator and phase shifter to control after DAC.

Assuming at time instant n, the signal to be transmitted is denoted as x(n), and $E[|x\left(n\right){|}^{2}]=1$. The vector $b_{t}$ represents the analog or digital transmit beamforming coefficients, and $b_{t}\in C^{J\times 1}$. The transmitter noise $n_{t}$ is complex additive white Gaussian noise (AWGN) with zero mean, and its covariance matrix is given by $N_{t}=E\left[n_{t}n_{t}^{H}\right]=\eta_{t}^{-1}Diag\left(b_{t}b_{t}^{H}\right)$, where $\eta_{t}$ represents the dynamic range or signal-to-noise ratio (SNR) of each transmit link, and Diag(·) is the function to get elements on the diagonal of a square matrix. As $b_{t}$ is one of the main focuses of this paper, its structure in the transmit link primarily consists of the components shown in Figure 5. In Figure 5, i denotes the corresponding transmit link index, with i=1,2,…,J. It just means that $b_{t,i}$ is the i-th element of $b_{t}$. An up-mixing, PA, and amplitude and phase control module, by digital or analog, should be contained.

For the composition of transmit beamforming including PA, the total transmit power $P_{t}$ is

(1) $$P_{t}=\sum_{i=1}^{J}P_{t,i}=\sum_{i=1}^{J}{\left|b_{t,i}\right|}^{2},$$

where $P_{t,i}$ represents the transmit power of the i-th transmit link.

Then, the signal vector at the transmit antennas in Figure 4 is

(2) $$t\left(n\right)=b_{t}x\left(n\right)+n_{t},t\left(n\right)\in C^{J\times 1}.$$

If the signals of interest (SoI) at the receive antennas are denoted as s(n), and the matrix $M\in C^{K\times J}$ describes the mutual coupling channels between the transmitting elements and the receiving elements, then the signal vector at the receiving antennas is given by

(3) $$\begin{array}{c} r\left(n\right)=s\left(n\right)+Mt\left(n\right)=s\left(n\right)+Mb_{t}x\left(n\right)+Mn_{t},r\left(n\right)\in C^{K\times 1}, \end{array}$$

where the mutual coupling channel matrix M is defined as

(4) $$M=\left[\begin{array}{ccc} S_{1,1} & \cdots & S_{1,J}\\ \vdots & \ddots & \vdots \\ S_{K,1} & \cdots & S_{K,J} \end{array}\right].$$

The coefficient $S_{k,j}$ represents the propagation coefficient between transmit element *j* and receive element *k*, after numbering each element of the transmitting and receiving apertures separately.

If we consider the phased array architecture for the receive array, we can define $b_{r}$ as the receive beamforming vector, where $b_{r}\in C^{K\times 1}$. The received noise $n_{r}$ is assumed to be a complex AWGN with a covariance matrix $N_{r}=E\left[n_{r}n_{r}^{H}\right]=\eta_{r}^{-1}Diag\left(E\left[rr^{H}\right]\right)+\sigma_{r}^{2}I$, where $\eta_{r}$ is the signal-to-noise ratio (or dynamic range) of each receive link, and $\sigma_{r}^{2}$ is the power of the receiver thermal noise floor. The received signal at the receiver without interference cancellation, denoted as *y*, is given by

(5) $$\begin{array}{c} y\left(n\right)=b_{r}^{H}\left[r\left(n\right)+n_{t}\left(n\right)\right]=b_{r}^{H}\left[s\left(n\right)+Mt\left(n\right)+n_{r}\left(n\right)\right], \end{array}$$

where $s\left(n\right)\in C^{K\times 1}$ is the external SoI, with a covariance matrix $R_{ss}=E\left[ss^{H}\right]$.

Typically, a STAR system includes a baseband digital SIC architecture. Let the coefficients of the digital interference cancellation filter be denoted as $c\to -b_{r}^{H}Mb_{t}$. Ideally, we can eliminate the correlated linear components of the transmitted signal x(n) from the self-interference at the digital receiver end, and the cancelled signal is

(6) $$\begin{array}{c} y^{\prime }\left(n\right)=y\left(n\right)+cx\left(n\right)=b_{r}^{H}\left[s\left(n\right)+Mn_{t}+n_{r}\left(n\right)\right]. \end{array}$$

Only transmit noise, nonlinearity, and receive noise are in the cancelled digital signal.

Note that, in this section, we assume the receiver is a digital phased array model with beamforming vector $b_{r}$, which is a typical example. But, in fact, the transmit beamforming method we proposed can be used in any multi-receiver scenarios.

### 2.2. Power Amplifier Model

Before presenting the optimization method of beamforming, the power amplifier model should be discussed first in this section, as a major limiting factor closely related to the transmit beamforming vector $b_{t}$.

**Figure 6:**
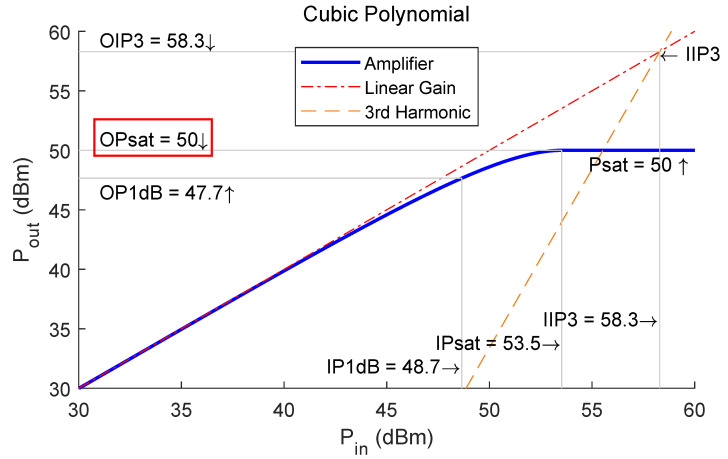
Output characteristic curve of a typical power amplifier with an OPsat of 50 dBm.

For the power amplifier used in the transmission link, we assume that all transmission links are uniform, which also implies that the same type of PA is used at the front end of each transmitter. In addition, the power amplification process of the PAs is typically nonlinear. Based on the Hammerstein polynomial model [23], its output characteristics (as an example) may be as shown in Figure 6.

Figure 6 shows a power amplifier type with a maximum output power (or saturated output power, OPsat) of 50 dBm (100 W), which also means that the saturated transmit power of this transmit channel is 50 dBm. This kind of PA with a high OPsat is usually used for the base stations and long-range detection radars. In practical applications, we need to consider various parameters of the PAs to meet the requirements of the transmitter. This non-linear characteristic of the PAs can be used as an optimization constraint for the next section on transmit beamforming optimization.

### 2.3. Transmit Beamforming Optimization

For improving the isolation between transmit arrays and each receive channel, we aim to minimize the incident SI power at each receive element. After the analysis of the STAR array architecture, how to optimize the transmit beamforming vector $b_{t}$ must be a major consideration. Here, we present the following optimization problem, P1:

(7) argminbtmax|Mbt|2,s.t.|bt,i|≤Pt,max,i=1,2,…,J,gt(ϕ,θ)×Re(qtHbt)≥γ,0<γ<Ptm.

The first line of this optimization problem presents the objective of optimizing the transmit beamforming vector, which is to minimize the maximum self-interference power incident at the receive elements from the transmitted signal x(n). It also notes that the transmit beamforming should adapt to the mutual coupling channels between the transmit array and each receive elements under some constraints, and suppress the maximum SI incident power above multiple receive elements as much as possible.

The second and third lines just provide the constraints for this problem. The second line incorporates the nonlinear power amplification characteristics of the power amplifier, assuming that all transmitting channels employ the same type of PA, and limits the maximum transmit power $P_{t,\max}$ on each link. This constraint tells us the total transmit power $P_{t}$ is not a constant value but is adjustable for Formula (1). In the third line, $g_{t}$ represents the radiation pattern of a single antenna of the transmit array, $q_{t}\in C^{J\times 1}$ is the transmit beamforming steering vector, and (ϕ,θ) denotes the desired pointing elevation angle and azimuth angle of the transmit array main beam. $P_{\mathrm{tm}}$ is the total transmit power when all transmit channels reach the saturation. It can be calculated as $P_{\mathrm{tm}}=P_{t,\max}J$ for the same OPsat of each channel. This line restricts the minimum radiated power of the transmit main beam in the desired direction to $\gamma^{2}$. It can avoid the high attenuation of transmit beam gain and the low level of transmit power, which causes the short transmission distance of radio signals.

Since multiple parameters in the aforementioned optimization problem are in a complex form, they are further simplified by separating them into real and imaginary parts:

(8) M=Re(M)+jIm(M),qt=Re(qt)+jIm(qt),bt=Re(bt)+jIm(bt).

Then, this optimization problem can convert to P2:

(9) $$\begin{array}{cc} \underset{b_{t}}{\arg\min}\max & |Mb_{t}{|}^{2},\\ s.t.\max(b_{t}^{\prime H}b_{t}^{\prime }) & \leq P_{t,\max},\\ q_{t1}^{\prime }b_{t}^{\prime }\geq \gamma /c, & 0<\gamma <\sqrt{P_{\mathrm{tm}}},\\ q_{t2}^{\prime }b_{t}^{\prime } & \leq \epsilon , \end{array}$$

where bt′=Re(bt)Im(bt), qt1′=Re(qtT),Im(qtT), qt2′=−Im(qtT),Re(qtT), $c=\sqrt{g_{t}\left(\phi ,\theta \right)}$.

In this problem, ϵ is a minimum value to prevent the imaginary part of the steering vector. The parameter $P_{t,\max}$ represents the maximum transmit power value that can be supported on each given transmission link, and γ denotes the minimum main beam gain in the desired angle (ϕ,θ). These two parameters can be set based on the actual system conditions and performance requirements.

If we disregard the variation of the antenna pattern with the scanning angle and primarily focus on the attenuation relative to the maximum gain at full transmit power, the constraint 2 in Formula (9) can be modified as follows:

(10) $$10\log_{10}\left(q_{t1}^{\prime }b_{t}^{\prime }\right)\geq 10\log_{10}\sqrt{P_{\mathrm{tm}}}-\gamma_{m}\left(\mathrm{dB}\right),\gamma_{m}>0\mathrm{dB},$$

where $\gamma_{m}$ represents the allowable relative maximum attenuation in dB. Note that we will use this constraint of attenuation during our simulation experiment in Section 3.2. This problem is a convex optimization problem, which can be solved using the CVX toolbox in Matlab (version: R2022a). Thus, we can obtain the optimized transmit beamforming vector and the maximum incident SI power at the receiving elements.

The above analysis was conducted under the assumption of narrowband coupling channels at the array elements. In reality, by supplementing the coupling matrix data at multiple frequency points within the signal bandwidth (which can be obtained through antenna simulations), this method can be extended to optimize the transmit beamforming for wideband signals. Specifically, the coupling matrices $M_{f_{i}}$ at multiple frequency points are used to construct the modified matrix $M^{\prime }$ with an increased number of rows.

(11) $$M^{\prime }=\left[\begin{array}{c} M_{f_{1}}\\ M_{f_{2}}\\ \vdots \\ M_{f_{N}}, \end{array}\right],$$

where $f_{1},f_{2},\ldots ,f_{N}$ represent N mutually uncorrelated frequency points within the signal bandwidth. It can be easily observed that $M^{\prime }\in C^{NK\times J}$. Then, this optimization problem can be reformulated as follows:

(12) $$\underset{b_{t}}{\arg\min}\max{\left|M^{\prime }b_{t}\right|}^{2}.$$

This problem aims to optimize the beamforming vector $b_{t}$ such that, at multiple frequency points, the maximum received power among all receiving elements is minimized. The constraints on $b_{t}$ optimization remain the same as discussed earlier in Formula (9).

As a transmit phased array system which can control the beam scanning by shifting the phase, the transmit beamforming vector should be calculated at each main beam pointing angle. In many practical scenarios, there should be a requirement for the scanning range of main beam maximum gain. Given this, the transmit beamforming design operation need to be repeated until the main beam pointing iterates through the entire range.

Finally, the algorithm complexity of the optimization problem P2 is primarily determined by the optimizer’s maximum number of iterations T and the number of columns in the coupling matrix M (or the number of transmit links) J. The algorithm complexity of the proposed transmit beamforming method is approximately $O(TJ^{3.5})$, indicating that the number of transmit channels in a STAR array has a significant impact on its performance. In the next subsection, we will analyze the effect of this method on the isolation performance of the STAR array.

### 2.4. System Isolation Analysis

For the simultaneous transmit and receive array system shown in Figure 4, due to the independence and uncorrelated nature of $x\left(n\right),s\left(n\right),n_{t}\left(n\right),$ and $n_{r}\left(n\right)$, we can compute the following power components at the receiver end without SIC.

The power of SoI received is:

(13) $$P_{y}^{s}=b_{r}^{H}R_{ss}b_{r}.$$

The SI power due to the transmitted signal is:

(14) $$P_{y}^{x}=b_{r}^{H}Mb_{t}b_{t}^{H}M^{H}b_{r}.$$

The SI power due to the transmitted noise is:

(15) $$P_{y}^{n_{t}}=b_{r}^{H}MN_{t}M^{H}b_{r}.$$

The power of the receiver noise is:

(16) $$P_{y}^{n_{r}}=b_{r}^{H}N_{r}b_{r}.$$

For these powers we presented, the resulting signal-to-interference-plus-noise ratio (SINR) of the received beam would be:

(17) $$\mathrm{SINR}=\frac{P_{y}^{s}}{P_{y}^{x}+P_{y}^{n_{t}}+P_{y}^{n_{r}}}.$$

To successfully achieve the simultaneous transmit and receive (STAR) operation, it is necessary to minimize these three power terms in the denominator as much as possible. By combining the optimized transmit beamforming method described in Section 2.3, the dominant component of the self-interference signal, $P_{y}^{x}$, will be significantly reduced. Due to the power limitation of the single-link transmission, $P_{y}^{n_{t}}$ will not change significantly, but it will still decrease slightly. Since most of the self-interference signal is suppressed at the receive antenna element, before the analog-to-digital conversion (ADC), the receiver noise power, $P_{y}^{n_{r}}$, will also decrease accordingly. Therefore, the SINR at the receiver will be greatly improved, enabling a successful simultaneous transmit and receive operation.

The above conjecture can also be verified by analyzing the covariance matrix $N_{r}$ of the receiver noise, which is

(18) $$\begin{array}{c} N_{r}=\eta_{r}^{-1}\mathrm{Diag}\left(R_{ss}\right)+\eta_{r}^{-1}\mathrm{Diag}\left(Mb_{t}b_{t}^{H}M^{H}\right)+\eta_{r}^{-1}\eta_{t}^{-1}\mathrm{Diag}\left(M\mathrm{Diag}\left(b_{t}b_{t}^{H}\right)M^{H}\right)+\sigma_{r}^{2}I. \end{array}$$

In the case of simultaneous transmission and reception, the self-interference power can be considered to occupy a significant portion of the incident power at each receiving element, so the power of the external desired signal can be neglected, yielding

(19) $$\begin{array}{c} N_{r}\approx \eta_{r}^{-1}\mathrm{Diag}\left(Mb_{t}b_{t}^{H}M^{H}\right)+\eta_{r}^{-1}\eta_{t}^{-1}\mathrm{Diag}\left(M\mathrm{Diag}\left(b_{t}b_{t}^{H}\right)M^{H}\right)+\sigma_{r}^{2}I. \end{array}$$

After the optimization of the transmit beamforming, both the first and second terms in Equation (19) will decrease, especially the first term, which corresponds to the eigenvalues of $N_{r}$. If we consider keeping the conventional receive beamforming unchanged, the receiver’s noise power will decrease.

The incident power $P_{r}$ at each receiving element is easily calculated as:

(20) $$P_{r}=\mathrm{Diag}\left(Mb_{t}b_{t}^{H}M^{H}\right)+\eta_{t}^{-1}\mathrm{Diag}\left(M\mathrm{Diag}\left(b_{t}b_{t}^{H}\right)M^{H}\right).$$

This is one of the most important indicators of a STAR array’s performance in this paper. By utilizing the objective function of the adaptive transmit beamforming method discussed in Section 2.3, the incident power $P_{r}$ at each receive element will be minimized within the given constraints. This approach effectively avoids receiver saturation and the limitations of the ADC dynamic range.

**Figure 7:**
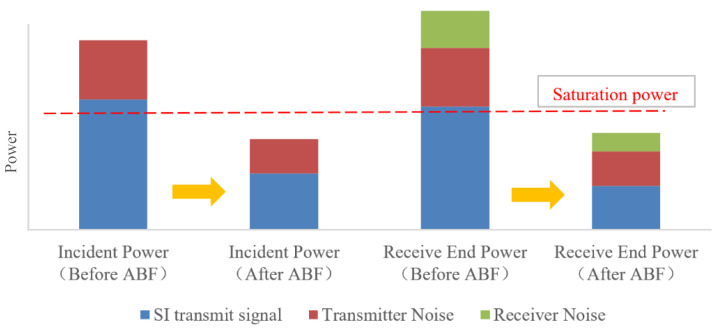
Stacked histogram of the power variations in SI and noise components at different parts of the receiver after ABF; the red dashed line represents saturated received incident power.

In conclusion, the variations in self-interference and noise components at different parts of the receiver after adaptive transmit beamforming can be referred to in the schematic diagram as shown in Figure 7.

For a STAR phased array system that requires the consideration of directional beam gain, we also need to measure the isolation performance using specific metrics, instead of using the traditional system isolation calculation $I=P_{n}/P_{t}$. The effective isotropic isolation (EII) of the radio system [24] can be defined as the ratio of the effective isotropic radiated power (EIRP) to the effective isotropic sensitivity (EIS):

(21) $$\mathrm{EII}=\frac{\mathrm{EIRP}}{\mathrm{EIS}},$$

where EIRP means the power required to provide equivalent illuminance in the desired direction can be defined on a phased array as follows:

(22) $$\begin{array}{c} \mathrm{EIRP}\left(\phi_{t},\theta_{t},b_{t}\right)=P_{t}G_{t}\left(\phi_{t},\theta_{t},b_{t}\right)=g_{t}\left(\phi_{t},\theta_{t}\right)b_{t}^{H}q_{t}\left(\phi_{t},\theta_{t}\right)q_{t}^{H}\left(\phi_{t},\theta_{t}\right)b_{t}, \end{array}$$

where $q_{t}\left(\phi_{t},\theta_{t}\right)=\exp\left\{-j2\pi \lambda \left(x_{t}\cos\left(\phi_{t}\right)\sin\left(\theta_{t}\right)+y_{t}\sin\left(\phi_{t}\right)\sin\left(\theta_{t}\right)\right)\right\}$ is the transmit steering vector. $P_{t}$ is the total power of the transmit array, and obviously $P_{t}=\sum_{i=1}^{J}P_{t,i}$. $G_{t}$ is the main beam gain of the transmit array. The variable $\phi_{t}$ represents the angle between the desired direction of the transmit beam on the array plane and the *x*-axis, while $\theta_{t}$ represents the angle between the beam direction and the *z*-axis. $x_{t}$ and $y_{t}$ denote the x and y coordinates of each transmitting antenna element on the array plane, respectively. $g_{t}\left(\phi ,\theta \right)$ represents the embedded element gain of the transmit array. As stated in Section 2.3, the amplitude constraint on the minimum radiated power of the transmit array (constraint 2) is equivalent to the constraint on the square root of minimum EIRP.

The EIS represents the thermal noise of a theoretical isotropic receiver with equivalent sensitivity in the desired direction. In phased array systems, it can be defined as

(23) $$\mathrm{EIS}=\frac{P_{n}}{G_{r}},$$

where $G_{r}$ is the gain of the receiving array, and $P_{n}$ is the residual SI and noise power at the receiving end. Furthermore, $G_{r}$ can be written as:

(24) $$G_{r}\left(\phi_{r},\theta_{r},b_{r}\right)=g_{r}\left(\phi_{r},\theta_{r}\right)b_{r}^{H}q_{r}\left(\phi_{r},\theta_{r}\right)q_{r}^{H}\left(\phi_{r},\theta_{r}\right)b_{r}.$$

The meanings of the symbols in Equation (24) are similar to those in the transmission case. Except for the positive sign in the complex exponential term, the definition of the receiving beam steering vector $q_{r}\left(\phi_{r},\theta_{r}\right)$ is the same as $q_{t}$, which is expressed as $q_{r}\left(\phi_{r},\theta_{r}\right)=\exp\left\{-j2\pi \lambda \left(x_{r}\cos\left(\phi_{r}\right)\sin\left(\theta_{r}\right)+y_{r}\sin\left(\phi_{r}\right)\sin\left(\theta_{r}\right)\right)\right\}$.

This difference between the steering vectors of the transmitting and receiving beams explains why the receive beamforming $b_{r}$ uses the conjugate transpose in signal flow analysis. Therefore, the calculation of EII can be rewritten as:

(25) $$\mathrm{EII}=G_{t}G_{r}I,$$

where $I=P_{t}/P_{n}$ is the traditional isolation calculation of a transceiver. From this formula, we note that EII is an effective index with which to measure both the isolation and array gain for a STAR array system.

The main task next is to calculate the total power $P_{n}$ of various self-interference and noise components at the receiving end. It can be written by the multiplication of the receive beamforming vector and noise covariance matrix $M_{b_{r}}$:

(26) $$P_{n}=b_{r}^{H}M_{b_{r}}b_{r},$$

where

(27) $$\begin{array}{c} M_{b_{r}}=Mb_{t}b_{t}^{H}M^{H}+\eta_{t}^{-1}M\mathrm{Diag}\left(b_{t}b_{t}^{H}\right)M^{H}+\eta_{r}^{-1}\mathrm{Diag}\left(Mb_{t}b_{t}^{H}M^{H}\right)\eta_{r}^{-1}\eta_{t}^{-1}\mathrm{Diag}\left(M\mathrm{Diag}\left(b_{t}b_{t}^{H}\right)M^{H}\right)+\sigma_{r}^{2}I. \end{array}$$

If we consider the introduction of the digital baseband self-interference cancellation (SIC) structure, which further reduces the self-interference power in the digital receiver based on the transmit beamforming optimization we proposed, the covariance matrix of the received self-interference noise is given by:

(28) $$\begin{array}{c} M_{b_{r}}^{\prime }=\eta_{t}^{-1}M\mathrm{Diag}\left(b_{t}b_{t}^{H}\right)M^{H}+\eta_{r}^{-1}\mathrm{Diag}\left(Mb_{t}b_{t}^{H}M^{H}\right)+\eta_{r}^{-1}\eta_{t}^{-1}\mathrm{Diag}\left(M\mathrm{Diag}\left(b_{t}b_{t}^{H}\right)M^{H}\right)+\sigma_{r}^{2}I. \end{array}$$

According to the matrix theorem $a^{H}\mathrm{Diag}\left({\mathrm{bb}}^{H}\right)a=b^{H}\mathrm{Diag}\left({\mathrm{aa}}^{H}\right)b$, Equation (27) can also be transformed into a quadratic form of the transmit beamforming vector:

(29) $$P_{n}=b_{t}^{H}M_{b_{t}}b_{t},$$

where

(30) $$\begin{array}{cc} M_{b_{t}} & =M^{H}b_{r}b_{r}^{H}M+\eta_{r}^{-1}M^{H}\mathrm{Diag}\left(b_{r}b_{r}^{H}\right)M+\eta_{t}^{-1}\mathrm{Diag}\left(Mb_{t}b_{t}^{H}M^{H}\right)+\eta_{r}^{-1}\eta_{t}^{-1}\mathrm{Diag}\left(M^{H}\mathrm{Diag}\left(b_{r}b_{r}^{H}\right)M\right)+\frac{\sigma_{r}^{2}}{P_{t}}I. \end{array}$$

Similarly, we also consider the case of introducing the digital baseband SIC structure, and Equation (30) should be changed to:

(31) $$\begin{array}{c} M_{b_{t}}^{\prime }=\eta_{r}^{-1}M^{H}\mathrm{Diag}\left(b_{r}b_{r}^{H}\right)M+\eta_{t}^{-1}\mathrm{Diag}\left(Mb_{t}b_{t}^{H}M^{H}\right)+\eta_{r}^{-1}\eta_{t}^{-1}\mathrm{Diag}\left(M^{H}\mathrm{Diag}\left(b_{r}b_{r}^{H}\right)M\right)+\frac{\sigma_{r}^{2}}{P_{t}}I. \end{array}$$

To simplify the analysis process and clarify the research direction, in the following analysis and simulation experiments, we consider the expected directions of the transmit and receive main beams to be the same, denoted as (ϕ,θ). By rearranging the above equations, we can obtain two equivalent expressions for calculating the EII of the STAR array as follows:

(32) $$\begin{array}{cc} \mathrm{EII} & =G_{r}\left(\phi ,\theta ,b_{r}\right)g_{t}\left(\phi ,\theta \right)\frac{b_{t}^{H}q_{t}\left(\phi ,\theta \right)q_{t}^{H}\left(\phi ,\theta \right)b_{t}}{b_{t}^{H}M_{b_{t}}b_{t}}\\ & =\mathrm{EIRP}\left(\phi ,\theta ,b_{t}\right)g_{r}\left(\phi ,\theta \right)\frac{b_{r}^{H}q_{r}\left(\phi ,\theta \right)q_{r}^{H}\left(\phi ,\theta \right)b_{r}}{b_{r}^{H}M_{b_{r}}b_{r}}. \end{array}$$

In the first line of Formula (32), the calculation of EII can be regarded as a generalized Rayleigh quotient in transmit beamforming $b_{t}$, if we hold $b_{r}$ constant. That will bring convenience for the EII calculation when $b_{t}$ is a parameter to be optimized.

Note that the previous calculation of EII is from the digital transmit end to the receive end of a STAR array, for using all the mutual coupling and receive beamforming information. It is easy to calculate the EII from the transmit array end to multiple receive elements or subarrays, which shows the positives of our method, by extracting specified rows of the mutual coupling matrix and the receive beamforming vector.

The following simulation experiments will demonstrate the effectiveness of the adaptive transmit beamforming method we proposed in improving the isolation between transmit and receive beams in the STAR array, using the metrics obtained in this section.

## 3. Simulation Results

To analyze the effect of our proposed transmit beamforming method on the STAR phased arrays, we design two kinds of ALSTAR array model in HFSS (version: 2020R2)at first, including an 8 Tx × 8 Rx uniform line array and a 16 Tx × 16 Rx uniform plane array. Then, we simulate the performance of ALSTAR models using the beamforming methods in MATLAB software (version: R2022a) with the coupling matrix and antenna parameters we got.

### 3.1. STAR Phased Arrays Design

**Figure 8:**
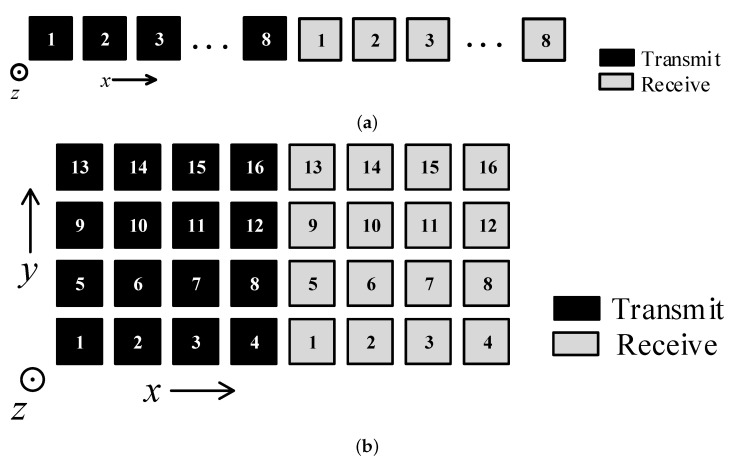
Layout of ALSTAR arrays: (**a**) 8 Tx × 8 Rx uniform line array; (**b**) 16 Tx × 16 Rx uniform plane array.

**Figure 9:**
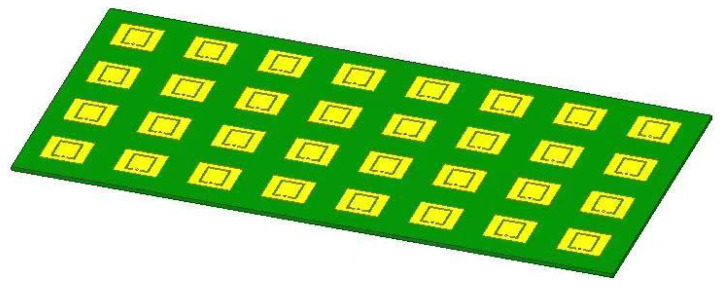
Antenna structure of 16 Tx × 16 Rx plane array. The same type of antenna unit was also used for the 16-element linear array.

The layout of these two arrays and the antenna structure are shown in Figure 8 and Figure 9.

In Figure 8, the number of the elements are also shown. The antenna type used by both these forms of the ALSTAR array is a linear-polarization U-slot antenna, which is shown in Figure 9 as an example of a 16 Tx × 16 Rx array. Both the parameters and the performance of this antenna are presented in our former study in detail [10]. The dielectric substrate utilized in this design is F4BM2-350 with a dielectric constant of 3.5, a loss tangent of 0.0017, and a thickness of 1 mm. The center frequencies of the antenna in these two arrays are 6 GHz and 4.5 GHz, and the operating bandwidth of the transmitted signal is 100 MHz. These two frequency ranges in C-band are usually used for detection and communication. The maximum gain of a single antenna reaches 5.5 dBi. The spacing between any two adjacent elements is half the wavelength of the center frequency.

For both the STAR systems, the transmit channel dynamic range is $\eta_{t}=45$ dB and the receive channel dynamic range is $\eta_{r}=70$ dB. The receive channels have thermal noise power $\sigma_{r}^{2}=-91$ dBm, which is calculated from the transmitted signal bandwidth.

Note that the STAR arrays we have shown are both ALSTAR arrays, but in fact this method is also suitable for the ELSTAR array system, as shown in Figure 1b, and we also performed an experiment based on it to observe an increase in isolation. For a more noticeable performance improvement observation, we just show the results based on these ALSTAR arrays.

### 3.2. System Performance Analysis

#### 3.2.1. Linear Array Simulation Experiment

Initially, we will demonstrate the effectiveness of the beamforming optimization on a one-dimensional linear array shown in Figure 8a for improving the isolation between transmission and reception, particularly when scanning only in the azimuthal direction (whose angle is represented by θ). Note that there are no additional SIC structures present in each of the STAR arrays during our experiment unless otherwise stated, for more obvious observation of the gain brought by the adaptive beamforming.

In this 8 Tx × 8 Rx array system, we assume that a PA with output characteristics as shown in Figure 6 is used at the front end of each transmit link. The maximum transmit power of a single channel is 100 W, resulting in a total maximum transmit power of 800 W. The fixed radiation power attenuation is limited to 3 dB. The scanning angle range for the main beam of this array is −45° to 45° in the azimuthal direction. In the simulation results, another commonly-used beamforming method [17] for STAR arrays is also plotted for comparison.

**Figure 10:**
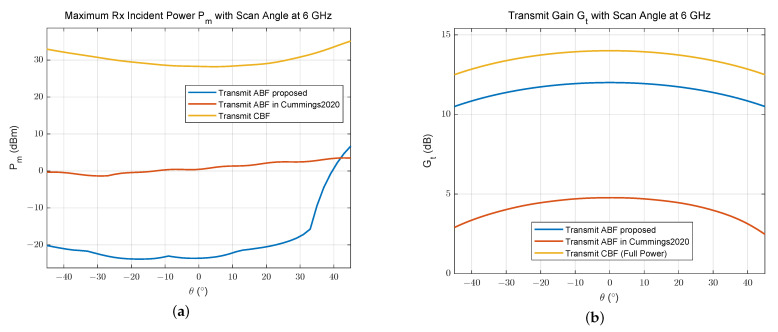
(**a**) Maximum receive incident power and (**b**) transmit gain under different beamforming for an 8 Tx × 8 Rx linear array [17].

Figure 10 shows that the maximum receive incident power above eight receive elements is reduced by about 50 dB within the scanning range after the transmit ABF we proposed, while that is only reduced by 30 dB after the ABF in [17]. In addition, the transmit array gain is only reduced by 3 dB after the proposed ABF, due to the constraint on the radiation power attenuation during optimization. This minor reduction in transmit gain ensures the proper functioning of the array system.

**Figure 11:**
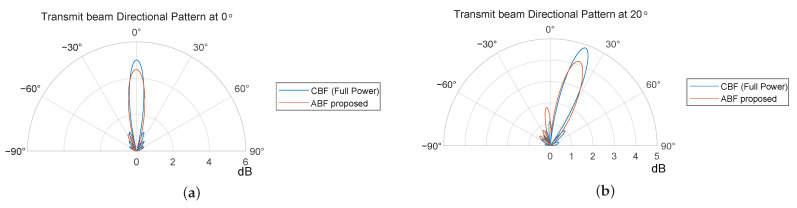
Transmit beam directional pattern (dB) pointed at (**a**) 0° and (**b**) 20° azimuth angle for an 8 Tx × 8 Rx linear array.

Figure 11 shows the alterations in the directional pattern of the transmit array when its main lobe is directed towards 0° and 20°. The reduction and broadening of its main lobe can be observed after transmit ABF. Due to the positioning of the transmit array on the left side of the receive array, there is a noticeable reduction in the amplitude of the sidelobes to the right of the main lobe, which effectively suppresses the ingress of SI into the receive channels.

**Figure 12:**
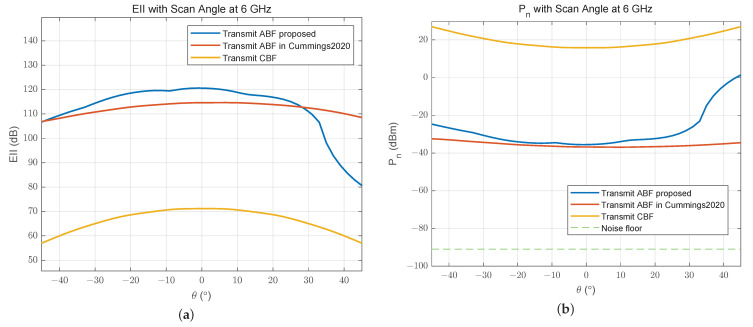
(**a**) End-to-end effective isotropic isolation and (**b**) residual noise power of receiver for an 8 Tx × 8 Rx linear array [17].

In Figure 12, the end-to-end EII improves by 50 dB at scan angle 0° after our proposed ABF, and it is slightly higher than that with the ABF in [17]. Within most scan angles (−45° to 32°), the EII has reached more than 106 dB. The residual noise power of receiver is reduced by 53 dB at most, which is very close to that of another method.

#### 3.2.2. Plane Array Simulation Experiment

Furthermore, to demonstrate the applicability and high performance of the beamforming method in a two-dimensional phased array with more receive channels, we conducted experimental validation on a 16-Tx and 16-Rx array system, as shown in Figure 8b. Its antenna array structure is presented in Figure 9. More performance outcomes will be discussed and analyzed below.

In this array system, the maximum transmit power of a single channel is 100 W, resulting in a total maximum transmit power of 1600 W. The scanning angle range for the main beam of this array is −60° to 60° in the azimuthal direction. The other parameters are the same as those in the 16-element linear array.

**Figure 13:**
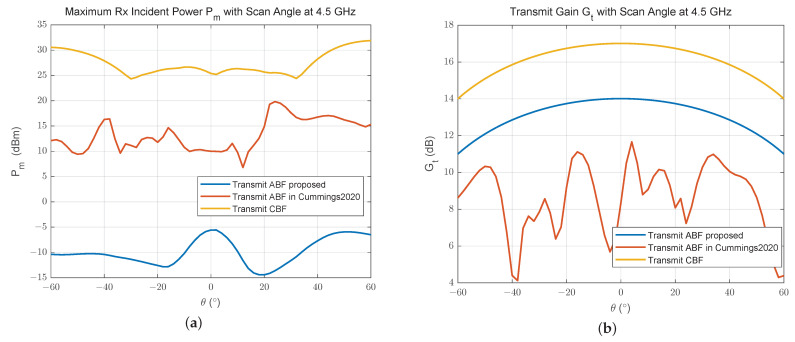
(**a**) Maximum receive incident power and (**b**) transmit gain under different beamforming for a 16 Tx × 16 Rx 2D array [17].

Figure 13 shows that the maximum receive incident power above 16 receive elements is reduced by about 30 dB within the scanning range after the transmit ABF we proposed, while that is only reduced by 15 dB after ABF in [17]. Additionally, the transmit array gain is only reduced by 3 dB after the ABF proposed, due to the constraint on the radiation power attenuation during optimization, while it is much lower and more undulating by using the method in [17].

**Figure 14:**
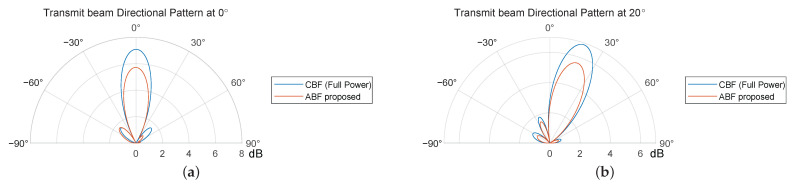
Transmit beam directional pattern (dB) pointed at (**a**) 0° and (**b**) 20° azimuth angle for a 16 Tx × 16 Rx array.

Figure 14 shows the alterations in the directional pattern of the transmit array when its main lobe is directed towards 0° and 20°. The reduction and broadening of its main lobe can be observed after transmit ABF. Due to the positioning of the transmit array on the left side of the receive array, there is a noticeable reduction in the amplitude of the sidelobes to the right of the main lobe, which effectively suppresses the ingress of SI into the receive channels. Compared to the directional pattern of the linear array shown in Figure 10, the width of the main lobe is widened for the fewer elements in the azimuthal direction.

**Figure 15:**
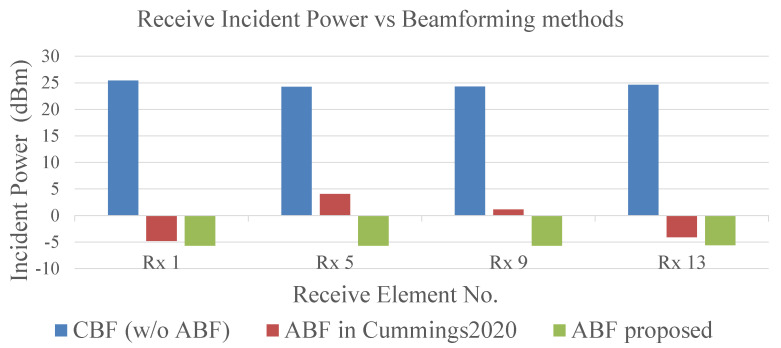
Each receive power under different beamforming methods for a row (No. 1, 5, 9, 13) in a 16 Tx × 16 Rx array [17].

Figure 15 shows the receive SI power of four receive elements, which is on the column closest to the transmit array. Compared to the other beamforming methods, the receive SI power of each element is at a minimum in the ABF we proposed, and its value is more stable and uniform at about −5.7 dBm. For the normalization of the receive array design, which usually depends on the maximum incident power, this result creates a looser requirement for the saturated incident power of receive links.

**Figure 16:**
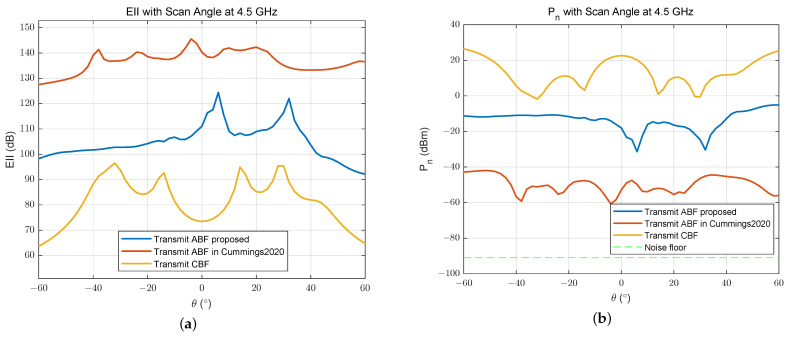
(**a**) End-to-end effective isotropic isolation and (**b**) residual noise power of receiver for a 16 Tx × 16 Rx array [17].

In Figure 16, we can see that the end-to-end EII with ABF in [17] is 30 dB higher than that with ABF we proposed, because the goal of that method is to maximize EII between the digital transmit end and the receive end. And the residual noise power at the receive end is lower than −40 dBm with that method. Compared to CBF, our ABF method still achieved a 35 dB improvement in EII when the scan angle is 0°. It seems that another method performs better in terms of the EII of a larger STAR array. But if we divide the receive array into two or more parts, for a flexible multifunctional subarray application, this result will be changed.

For a clear comparison, Table 2 summarizes the previous performance under different transmit beamformings in two kinds of STAR array systems.

**Figure 17:**
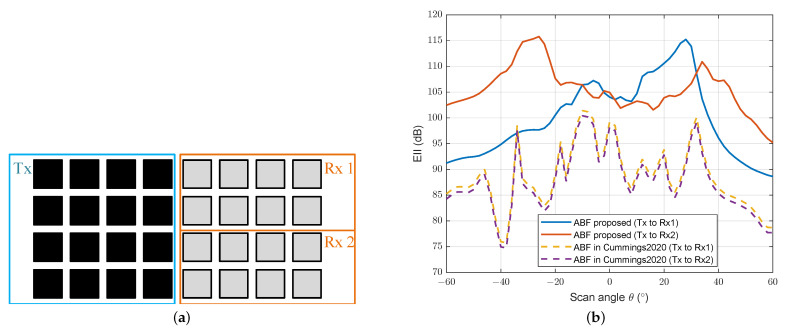
(**a**) Subarray partition for a 16 Tx × 16 Rx array; (**b**) effective isotropic isolation between the transmit array and each receive subarray with various beamforming methods [17].

Additionally, the transmit ABF method proposed will bring benefits in multifunctional array applications. In Figure 17a, we divide the receive array into two parts, and there are different uses for them, such as communication and position. Figure 17b just shows the EIIs between the transmit array and each receive subarray with the beamforming methods mentioned before.

After the division of the receive array, the ABF method we proposed performs better than that in [17] in terms of EII between Tx and each Rx. Because our method tries to improve the isolation between the transmit array and each receive element, no matter how the array divides, the EII will be maintained at a high level. This suggests that our method is more suitable for the multifunctional STAR subarray technology.

**Figure 18:**
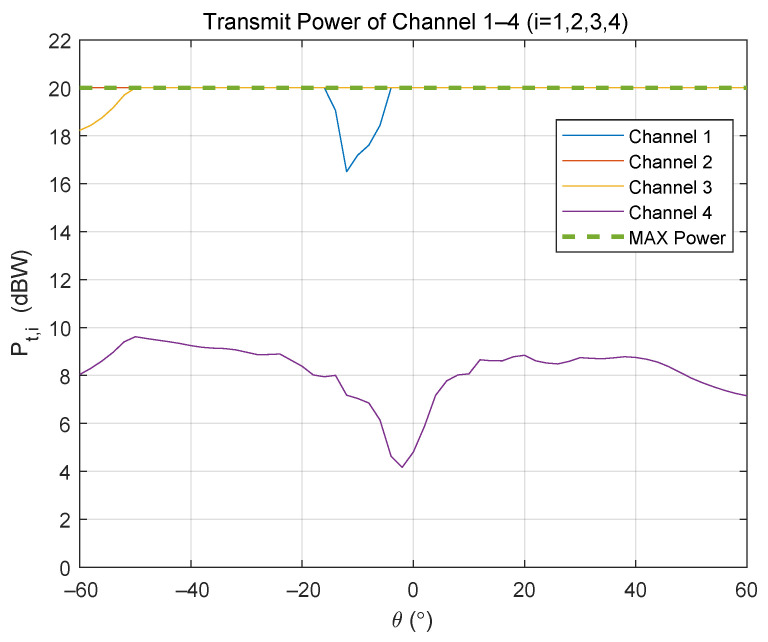
The variation of single transmit power with scanning angle for a row in a 16 Tx × 16 Rx array. The numbers of channels are located at the bottom row of the transmit array.

**Figure 19:**
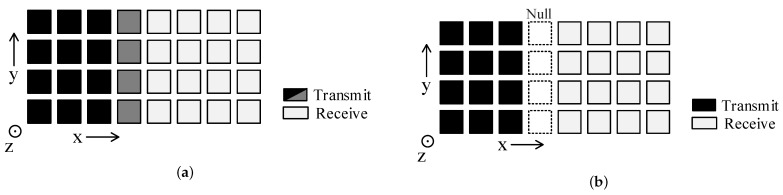
Application of the irregular transmit array in a 16 Tx × 16 Rx 2D ALSTAR array: (**a**) partial low-power; (**b**) null-element.

Figure 18 shows the transmit power of the transmit channels in the bottom row of the 16-channel transmit array. In fact, the other rows’ transmit power variation is similar. Within the entire scanning range, the transmit power of Channel 4, which is closest to the receive array, keeps a low level of about 10 W. In addition, the transmit power of the other three channels is at or near the maximum. It means that the PAs in these channels almost work in the saturation region, as shown in Figure 6. Therefore, the PAs in Channels 1 to 3 are used almost fully, while that in Channel 4 is not visibly. That suggests an irregular structure for simplifying the transmit array, as shown in Figure 19.

In this irregular transmit array structure, the difference is that the elements and channels on the fourth column (which is highlighted in gray) are replaced to a lower-power version. The acceptable maximum transmit power standard of these analog components and antennas reduces to 10 W, which can reduce the cost of designing the array effectively. If we ignore the gain bought by the low-power channels and aim to save more, we can remove the fourth column directly, which is called null-element. This example can also expand to larger ALSTAR arrays with more channels, and certainly save costs on the array design.

Next, we will discuss the effect of ADC quantizer bits in receive links. The common ADC quantization bit depths are 8, 12, and 16 bits, including the sign bit. With the calculation of the minimum SoI power, if the incident SoI power is even lower than it, the ADC in this receive link will not be able to quantize the analog SoI signal. For this, even after digital SIC, the desired weak digital SoI signal cannot be obtained. Using the above-mentioned beamforming method to obtain the receive maximum incident power, we can calculate the minimum quantifiable SoI power $P_{SoI}$ for the ADC as follows:

(33) $$P_{\mathrm{SoI}}=P_{\mathrm{SI}}\times {\left(\frac{1}{2^{B-1}}\right)}^{2},$$

where *B* represents the ADC quantization bit, $P_{\mathrm{SI}}$ represents the maximum incident receive power above all receive elements. Then, Table 3 shows the minimum quantized SoI power in dBm of three kinds of ADCs by using different beamforming methods.

We can see that, after using the ABF method we proposed, the minimum quantized SoI power reaches −96.0 dBm with a 16-bit ADC quantizer, which is common in most application scenarios. Additionally, if we reduce the number of quantizer bits to 12, the minimum quantized power is −71.9 dBm, even lower than that using a 16-bit ADC quantizer and the ABF method in [17]. Combined with the discussion about Figure 15, if we have estimated the minimum power of SoI that needs to be quantified, we will obtain fewer-bit ADCs for the digital receiver design.

Further, the power of SI and noise need to be suppressed more at the receiving digital end, to detect the low-power SoI successfully. The digital SIC structure and receive ABF (when working as a receive phased array mode) can be used, as shown in Figure 4. There are many studies about these, so we will not discuss these in this paper anymore.

## 4. Conclusions

In this paper, we propose an optimization method for transmit beamforming based on maximum transmit power and minimum radiation power, specifically targeting the STAR phased array. The objective is to minimize the incident self-interference power on each receive channel. Through simulation experiments, it has been observed that, within the beam scanning range of a 16 Tx × 16 Rx 2D plane ALSTAR array, the maximum incident power on each receive channel is reduced by more than 30 dB. This effectively mitigates the loss of signals of interest caused by finite-bit ADCs and prevents receiver saturation, thereby enabling other self-interference suppression techniques, such as the digital domain SIC, to function properly. Moreover, this method allows direct control over the radiation power of the main beam or the attenuation of the array gain, avoiding significant attenuation caused by adaptive beamforming. Additionally, the proposed transmit beamforming method simplifies the design of transmit links in the ALSTAR array system, reducing costs by incorporating low-power standard analog devices in some of them, which is called an irregular transmit array, as opposed to a uniform standard design for the entire transmit array as is traditionally done. Future work will mainly focus on implementing this transmit ABF method on FPGA/DSP, and isolation analysis adding digital SIC or receive ABF technology. Furthermore, the array prototypes will be manufactured for testing and verification.

## Figures and Tables

**Table 1:** Table of acronyms.

Acronym	Definition
STAR	Simultaneous Transmit and Receive
IBFD	In-band Full-duplex
FPGA	Field Programmable Gate Array
DSP	Digital Signal Processor
STAR	Simultaneous Transmit and Receive
ALSTAR	Aperture-Level Simultaneous Transmit and Receive
ELSTAR	Element-Level Simultaneous Transmit and Receive
MIMO	Multiple Input and Multiple Output
SoI	Signal of Interest
FDD	Frequency Division Duplex
TDD	Time Division Duplex
SI	Self-Interference
SIC	Self-Interference Cancellation
ADC/DAC	Analog-to-Digital Converter/Digital-to-Analog Converter
PA	Power Amplifier
SNR	Signal and Noise power Rate
SINR	Signal-to-Interference-plus-Noise power Rate
AWGN	Additive White Gaussian Noise
OPsat	Output Power of Saturation
EII	Effective Isotropic Isolation
EIRP	Effective Isotropic Radiated Power
EIS	Effective Isotropic Sensitivity
ABF	Adaptive Beamforming
CBF	Conventional Beamforming (Only Phase-Shift)

**Table 2:** The performance of STAR array systems within the scanning range.

Array Type	Beamforming Method	EII (dB)	Receive Incident Power (dBm)	Transmit Gain Attenuation (dB)
16-element Linear Array	CBF	71.2/57.0/66.3	35.0/28.2/30.2	No attenuation
	ABF in [17]	114.6/106.8/112.3	3.8/−1.3/0.9	10.8/9.2/9.5
	ABF proposed	120.6/80.6/112.4	6.7/−23.8/−19.0	3.0/3.0/3.0
32-element Plane Array	CBF	96.3/63.6/81.8	31.8/24.3/27.4	No attenuation
	ABF in [17]	145.1/127.5/136.6	19.8/6.6/13.0	12.2/5.1/7.7
	ABF proposed	124.9/92.2/105.0	−5.7/−14.4/−9.9	3.0/3.0/3.0

The performance indicators are shown in maximum/minimum/average values.

**Table 3:** The number of ADC quantizer bits versus the minimum power of the quantized SoI.

Beamforming Method	Maximum Incident Receive Power (dBm)	Number of ADC Quantizer Bits	Minimum Power of Quantized SoI (dBm)
CBF	31.84	8	−10.30
		12	−34.39
		16	−58.47
ABF in [17]	19.83	8	−22.31
		12	−46.40
		16	−70.48
ABF proposed	−5.70	8	−47.80
		12	−71.90
		16	−96.00

## Data Availability

Data are contained within the article.
